# Function-Focused Care and Its Association With Adverse Events

**DOI:** 10.1093/geroni/igaf122.722

**Published:** 2025-12-31

**Authors:** Marie Boltz

**Affiliations:** Pennsylvania State University, University Park, Pennsylvania, United States

## Abstract

Although the benefits of function-focused care (FFC) are well known, its implementations continues to be slow to take hold in the hospital setting, in part due to concerns for patient safety. Clinicians, patients, and families have expressed worry about the potential for falls and fall-related injuries which is associated with increased hospitalizations and mortality. This study addressed that issue by examining whether FFC was associated with increased falls, transfers to the hospital, and hospital readmission. Mixed-effects logistic regression demonstrated that, adjusting for race, age, co-morbidity, discharge location, and dementia severity, hospitalized older adults in the intervention arm had no more falls at one ( p=.512) and six (p= .668) months as compared to those in the control (education-only) arm. Findings suggest that, contrary to commonly held beliefs, FFC can be implemented without concern for increasing falls or risk of emergency room visits or hospitalizations.

